# Mutual association of *Broad bean wilt virus* 2 VP37-derived tubules and plasmodesmata obtained from cytological observation

**DOI:** 10.1038/srep21552

**Published:** 2016-02-23

**Authors:** Li Xie, Weina Shang, Chengke Liu, Qinfen Zhang, Garry Sunter, Jian Hong, Xueping Zhou

**Affiliations:** 1 State Key Laboratory of Rice Biology, Institute of Biotechnology, Zhejiang University, Hangzhou 310058, China; 2 Zhejiang Academy of Agricultural Sciences, Hangzhou 310021, China; 3 State Key Laboratory of Biocontrol, School of Life Science, Sun Yat-Sen University, Guangzhou 510275, China; 4 Department of Biology, University of Texas at San Antonio, San Antonio TX 78249, USA; 5 Center of Analysis and Measurement, Zhejiang University, Hangzhou 310029, China

## Abstract

The movement protein VP37 of broad bean wilt virus 2 (BBWV 2) forms tubules in the plasmodesmata (PD) for the transport of virions between cells. This paper reports a mutual association between the BBWV 2 VP37-tubule complex and PD at the cytological level as determined by transmission electron microscopy. The generation of VP37-tubules within different PD leads to a different occurrence frequency as well as different morphology lines of virus-like particles. In addition, the frequency of VP37-tubules was different between PD found at different cellular interfaces, as well as between single-lined PD and branched PD. VP37-tubule generation also induced structural alterations of PD as well as modifications to the cell wall (CW) in the vicinity of the PD. A structural comparison using three-dimensional (3D) electron tomography (ET), determined that desmotubule structures found in the center of normal PD were absent in PD containing VP37-tubules. Using gold labeling, modification of the CW by callose deposition and cellulose reduction was observable on PD containing VP37-tubule. These cytological observations provide evidence of a mutual association of MP-derived tubules and PD in a natural host, improving our fundamental understanding of interactions between viral MP and PD that result in intercellular movement of virus particles.

The surrounding of a plant cell by a cell wall (CW) results in segregation of the symplasm. To maintain a connection between the symplasm and allow transport of materials between cells, a size adjustable gateway, plasmodesmata (PD), is present on the CW^1^. The desmotubule (DT) located in the center of PD functions as a symplasm connecting bundle, composed of appressed endoplasmic reticulum (ER) and the cytoskeleton^2^. The remaining spaces around the DT (cytoplasmic sleeves) are sustained by several protein spokes, and is used for transportation^3^,^4^. These structural features place a limit on the size of materials that can be transported, defined as the size exclusion limit (SEL)^1^. The permeability of PD can be adjusted as demonstrated through protein interactions^1^ along with turnover of callose deposition nearby^5^. In addition, PD can be divided into primary PD (de novo generated), which exhibit a single-lined morphology in sink tissue and secondary PD, which show both single-lined and branch morphologies in source tissue^3^,^6^,^7^. The biogenesis of secondary PD originates from the primary PD post-cytokinesis^8^.

Establishment of a plant virus infection requires both intercellular and long distance movement. For intercellular movement, viruses face the CW, which can act as a barrier. To pass through this barrier, viruses encode a movement protein (MP) that can interact with other viral or host factors at the PD^9^,^10^,^11^,^12^. As a result, the PD undergo structural alterations that result in a larger SEL. Different viruses utilize different movement protein-PD interactions for intercellular movement. We can divide the structural alterations of PD into the ‘MP-RNA’ pattern (exemplified by tobacco mosaic virus, TMV) and the ‘tubule-guided’ pattern (exemplified by cowpea mosaic virus, CPMV)^11^,^13^. During TMV movement, in which only the viral genome is transported, MP specifically localizes to the secondary PD^14^. This leads to a slight increase in the SEL without any obvious ultra-structural change^13^. For CPMV infection, MP induces formation of tubules in the PD, which results in substantial ultra-structural changes, including the absence of DT and large increases in the SEL^15^. The tubule functions as the guide for transporting the spherical virion through the PD^16^,^17^. In ‘tubule-guided’ virus movement, host factors such as Plasmodesmata Located Protein (PDLP) acts as a PD recognition receptor by interacting with viral MP^18^.

Broad bean wilt virus 2 (BBWV 2) is a member of the *Fabavirus* genus, family *Comoviridae,* and employs the ‘tubule-guided’ pattern for intercellular movement^19^. Interestingly, there are two types of BBWV 2-associated tubular structures that can be observed by transmission electron microscopy (TEM) in infected *Chenopodium quinoa*. One tubule is 50 nm in diameter and distributed within the PD. The 37 kDa viral protein (VP37) encoded by RNA2^20^, is the MP of BBWV 2^21^, and was identified as the major component of this tubule^19^. For this paper, we will use the tentative name ‘VP37-tubule’ to describe this kind of tubule. The VP37-tubule guided virus movement of BBWV 2 resembles that of CPMV. The second tubule has a diameter of 80 nm and is composed of virions. It is located in the cytoplasm, and exhibits no relationship with the PD^19^. As observed in the natural host of *C. quinoa*, the VP37-tubule can assemble in both plant protoplasts and insect cells, which revealed that the key factors for tubule formation are conserved in plants and animals^19^. So far, the studies on ‘tubule-guided’ intercellular movement have mainly focused on the MP-derived tubule with less emphasis on the relationship between the tubule and the PD. In this study, we observed that the PD at different cellular interfaces influenced the frequency of VP37-tubules, and that the presence of VP37-tubules led to structural alterations of the PD, and CW modifications. These results increase our fundamental understanding of the interactions between MP-tubules and the PD.

## Results

### Morphology of VP37-tubules in PD

**Figure 1:**
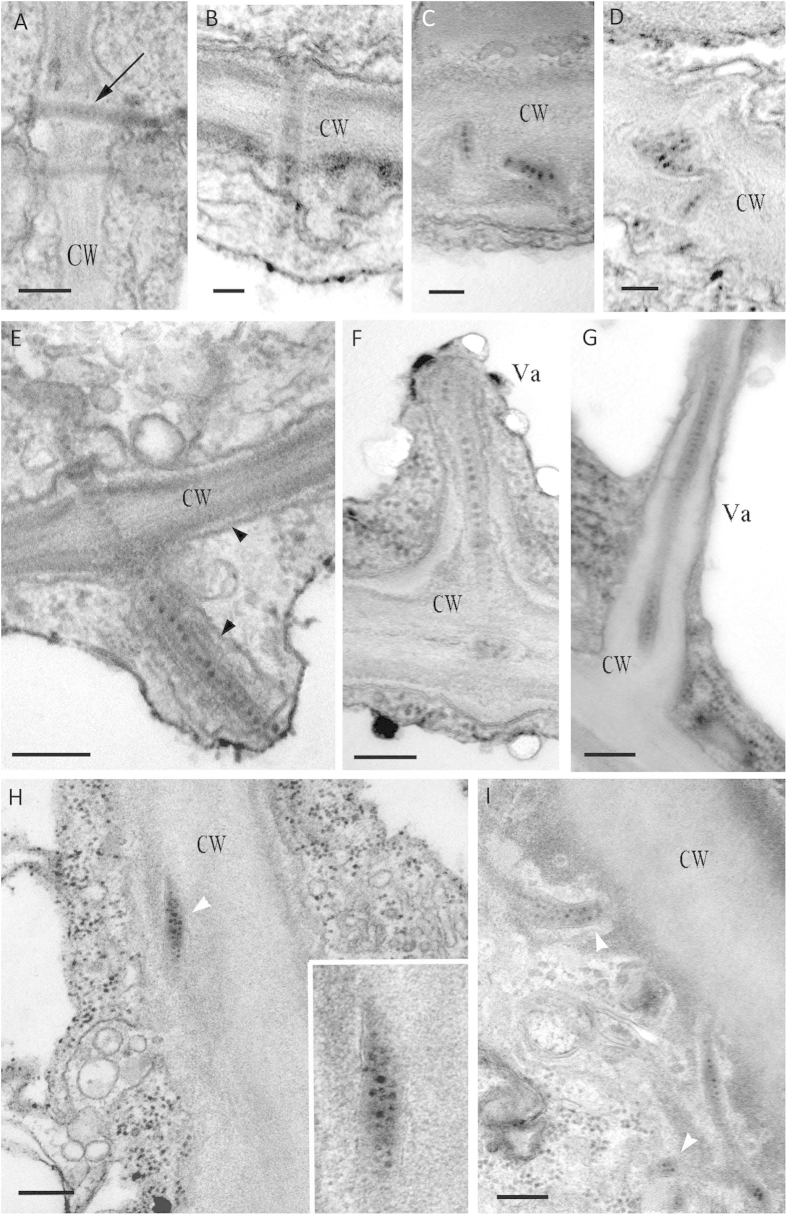
Morphology of VP37-tubules in broad bean wilt virus 2 infected leaves. (**A**) Size comparison between plasmodesmata (PD) containing or lacking VP37-tubules. The tubule is marked with an arrow. Bar = 200 nm. (**B–D**) The VP37-tubule with a branched morphology was observed in the branched PD. Bar = 100 nm. (**E–G**) VP37-tubules elongated from PD to cytoplasm or even attached to vacuole. Cell wall (CW) material as well as the cellular membrane (black arrowhead) was expanded and protruded around the tubule. Bar = 200 nm. (**H,I**) The VP37-tubule with a widened morphology is packaged with multiple lines of VLPs (white arrowhead), and was observed in PD and CW-adjacent cytoplasm. Bar = 200 nm. Va = vacuole.

In *C. quinoa* leaves inoculated with BBWV 2, VP37-tubules were mostly observed in PD (Fig. 1). In the presence of VP37-tubules we observed enlargement of the PD, with a diameter of 50–60 nm (Fig. 1A, black arrow), as compared to normal PD (20–30 nm). The typical pattern of a VP37-tubule was single stranded with virus-like particles (VLP) packaged inside, whose structure approximately matched that of the PD in which it resided (Fig. 1A). Apart from this typical morphology, we also observed some tubules that exhibited diverse unusual morphologies, which have not been highlighted in previous reports. Some VP37-tubules were observed in a branched configuration (Fig. 1B,C), very similar to the structure of secondary PD, or were located in the central cavity of secondary PD (Fig. 1D). Some of the VP37-tubules found within the secondary PD were observed to have extensive elongation into the cytoplasm, and in some cases as far as the vacuolar membrane (Fig. 1E–G). Elongation of the tubule from the CW to the cytoplasm appeared to cause an increase in peripheral CW materials and the cellular membrane that protruded around the tubule (Fig. 1E, black arrowhead). Some VP37-tubules located within the PD were wider or more closely associated with the CW. Within the tubules, multiple lines of VLPs were packaged in tandem (Fig. 1H,I, white arrowhead).

### Distribution and frequency of VP37-tubules in different PD

**Figure 2:**
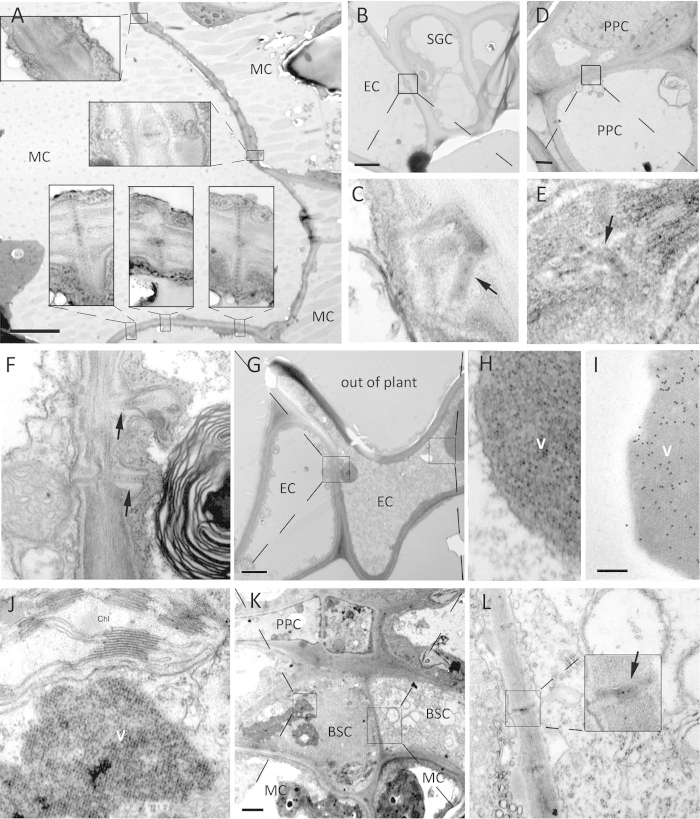
VP37-tubules are located on different cellular interfaces. (**A**) Tubules were observed on the mesophyll-mesophyll interfaces. Bar = 5μm. (**B,C**) Tubule-like structures were observed on the interface of epidermal-safe guard cell. Bar = 2μm. (**D–E**) Tubule-like structures were observed on the interface of phloem parenchyma cell-phloem parenchyma cell. Bar = 1μm. (**F–H**) Tubule-like structures were observed on the cellular interface of epidermal cells. Bar = 2μm. In that, (**F**) was the amplified image of tubule and (**H**) was the amplified image of virus crystal-like structure. (**I**) Immuno-gold labeling of crystals of broad bean wilt virus 2 particles. Bar = 200nm. (**J–L**) Tubule-like structures were observed on the cellular interface of bundle sheath cells. In that, (**L**) was the amplified image of tubule and (**J**) was the amplified image of virus crystal-like structure. Bar = 2μm. MC = mesophyll cell, EC = epidermal cell, SGC = safe guard cell, PPC = phloem parenchyma cell, BSC = bundle sheath cell, V = crystal of virus particles.

The study of the relationship between VP37-tubules and PD was obtained from systemically infected *C. quinoa* leaves. In our cytology studies, it was observed that not all PD contained VP37-tubules, and that the number of VP37-tubule-PD complexes detected in cells varied (Fig. 2). These observations imply that the distribution of VP37-tubules in PD is heterogeneous. What was less clear, however, was whether a difference in VP37-tubule frequency or distribution existed. Therefore, we compared the frequency of VP37-tubules in PD between different cellular interfaces and between single-lined or branched PD. Under TEM, VLP accumulation in cells as well as the generation of VP37-tubules in PD was observed, and a total of 155 VP37-tubules were observed in 40 sections prepared from *C. quinoa* leaves (Table 1).

A difference in the frequency of VP37-tubules associated with different types of cellular interface was observed in the leaves of BBWV 2 infected *C. quinoa*. VP37-tubules were frequently observed in PD at the mesophyll cell (MC)-MC interface (Fig. 2A and Table 1), while a small percentage of VP37-tubules (20%) were also observed at cellular interfaces different to the MC-MC interface. VP37-tubules were distributed in PD on the CW interfaces between epidermal cell (EC)-safe guard cell (SGC) (Fig. 2B,C), phloem parenchyma cell (PPC)-PPC (Fig. 2D,E), EC-EC (Fig. 2F–H) and bundle sheath cell (BSC)-BSC (Fig. 2J–L). Analysis of the morphology of VP37-tubules resident in the PD of SGC, PPC and BSC revealed a short, narrow structure that contained more condensed material (Fig. 2C,E,L). The morphology of VP37-tubules resident in the PD of EC resembled that observed in MC. Moreover, in the cytoplasm of EC (Fig. 2H) and BSC (Fig. 2J), we also observed crystal-like structures comprised of BBWV 2 virions, which was shown by immuno-gold labeling (Fig. 2I). So far, we have not observed that distribution of VP37-tubules at the cellular interfaces of other kinds of cells.

In addition to assess the distribution of VP37-tubules on different cellular interfaces, we tested whether there were any differences in the frequency of VP37-tubules present on the diverse cellular interfaces. To do this we used the parameter Occu-F_tubule_, which is a measure of the occurrence of VP37-tubules in PD without taking into account the frequency of PD (F_PD_) found in different cell types. A second measurement, Dist-F_tubule_, was used to indicate the distribution of VP37-tubules on a particular cellular interface, which integrates any differences in F_PD_. For this we classified the cellular interfaces into three major groups, including the mesophyll group (MC-MC interface), epidermis group (EC-EC and EC-SGC interface) and phloem group (PPC-PPC and BSC-BSC interface). The frequency of VP37-tubules and PD in these tissues was calculated from the average of 20 randomly selected TEM fields. The data for each cellular interface group, in three independent replicates, was compared, and illustrated that the frequency of VP37-tubules (both Occu-F_tubule_ and Dist-F_tubule_) was highest in the epidermis group, and lowest in the phloem group, with an intermediate VP37-tubule frequency in the mesophyll group (Table 2). Based on these results, we conclude that BBWV 2 infection leads to the formation of VP37-derived tubules, which associate with PD at different cell interfaces. This suggests that BBWV 2 may utilize VP37-tubules formed within PD as a pathway for intercellular transport of the viral genome in leaf epidermis, mesophyll and phloem tissue. Although we observed a greater frequency of VP37-tubules associated with PD in the epidermis as compared to mesophyll or phloem, we do not know how this correlates with BBWV 2 movement between the various cell interfaces.

We next tested whether we could detect any differences in the frequency of VP37-tubules present in single-lined versus branched PD. For this, the numbers of either single-lined or branched PD present in all cell types were pooled based on morphology. Out of a total of 155 VP37-tubules that were detected, 75 were found to be associated with single-lined PD, and 80 with branched PD. For the comparison we counted the number of VP37-tubules, number of PD, and measured CW length in 20 randomly selected regions that included epidermal, mesophyll and phloem cells. This was repeated three times (Table 3). As can be seen, the frequency of single-lined PD was approximately two-fold lower than the frequency of branched PD. VP37-tubules were more likely to be associated with single-lined PD than branched PD, as indicted by a higher Occu-F_tubule_ (Table 3). This suggests that VP37-tubules are able to form in both single-lined and branched PD but with a higher frequency in single-lined PD.

### Structural alteration of PD by VP37-tubules

**Figure 3:**
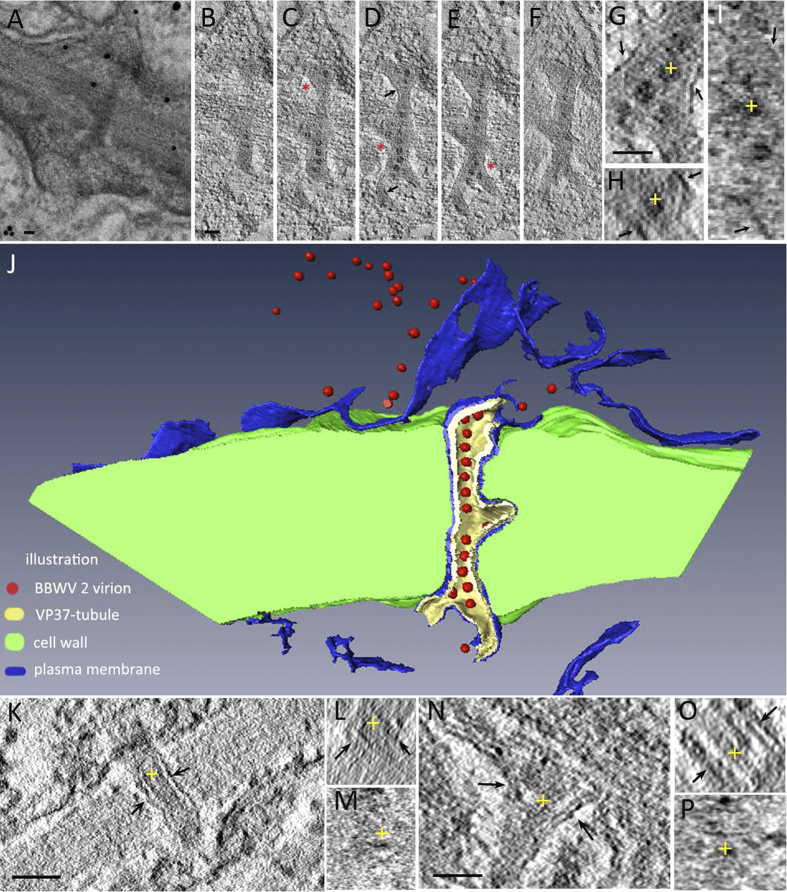
Structural comparison of plasmodesmata (PD) associated with VP37-tubules and virus-free PD. The membrane-like structure attached to the VP37-tubule is marked with an arrow. (**A**) A projection image of broad bean wilt virus 2 VP37-tubules in PD taken by conventional TEM. Bar = 50nm. (**B–J**) The desmotubule (DT)-like structure is absent in the three dimensional (3D) structural model of VP37-tubules. Bar = 50nm. (**B–F**) Digital slices of the structural model (total thickness = 105 nm) at the z height of 60nm, 67nm, 77nm, 84nm and 93nm respectively. Cell wall (CW) modification observed with PD associated with VP37-tubules is marked with a red asterisk. (**G–I**) The structural model was observed from three dimensions (XY, XZ and YZ). The structural detail at the same position from three dimensions was marked with a yellow cross. Bar = 50nm. (**J**) The structural model of PD associated with VP37-tubule was rendered with pseudo color, which was segmented from 3D density map. (**K–M**) A DT-like structure was observed in the 3D structural model of virus-free, single-lined PD. The DT shown at the same position in each of the three dimensions is marked with a yellow cross. Bar = 50nm. (**N–P**) A DT-like structure is observed in the 3D structural model of virus-free, branched PD. The DT shown at the same position in each of the three dimensions is marked with a yellow cross. Bar = 50nm.

The presence of VP37-tubules in PD was associated with obvious structural alterations of the PD, observable under TEM. However, some of the structural details were concealed when we performed a structure overlay of a 2D projection on a conventional TEM image. As this could lead to false interpretation^22^, we performed electron tomography (ET) to generate reconstructed tomograms of 3D density map (Fig. 3A–I). By using 3D ET, we made a comparison between the structure of virus-free PD (both single-lined and branched PD) and VP37-tubule associated PD. The most prominent difference between the two types of PD was the expanded size of the VP37-tubule associated PD and presence of a DT-like structure in the center of virus-free PD, which was visible as an electron dense bundle in the XY dimension (Fig. 3K). A membrane-like structure that was wrapped around virus-free PD was also detectable (Fig. 3K arrow). The DT-like bundle and membrane-like structure could also be observed in the other two dimensions (YZ and XZ) (Fig. 3L,M, membrane was marked as arrow). In Fig. 3, the yellow cross represents the identical position in the structural model as observed from each of the three dimensions. A similar DT-like structure with a cavity was also observed in secondary PD in all three dimensions (Fig. 3N–P). In contrast, the DT-like structure was absent in PD containing VP37-tubules (Fig. 3B–F). Observations from all three dimensions confirmed the absence of the DT-like structure, with only VLPs present (Fig. 3G–I). A membrane-like structure could also be observed around the VP37-tubules. Furthermore, presence of the VP37-tubule correlated with an enlargement of the PD diameter as compared to the PD that did not contain tubules. Based on measurements from 30 samples, the diameter of virus-free PD was an average of 28.40 nm (Standard deviation of ±1.19), while the diameter of PD containing VP37-tubules was an average of 53.14 nm (Standard deviation of ±2.68). Using the 3D tomograms, we segmented the PD containing VP37-tubules and surface rendered it as a pseudo-colored model to reveal the stereo profile. From this model, absence of the DT-like structure and presence of VLPs in PD associated with VP37-tubules could easily be distinguished (Fig. 3J).

### Alteration of CW components in PD associated with VP37-tubules

**Figure 4:**
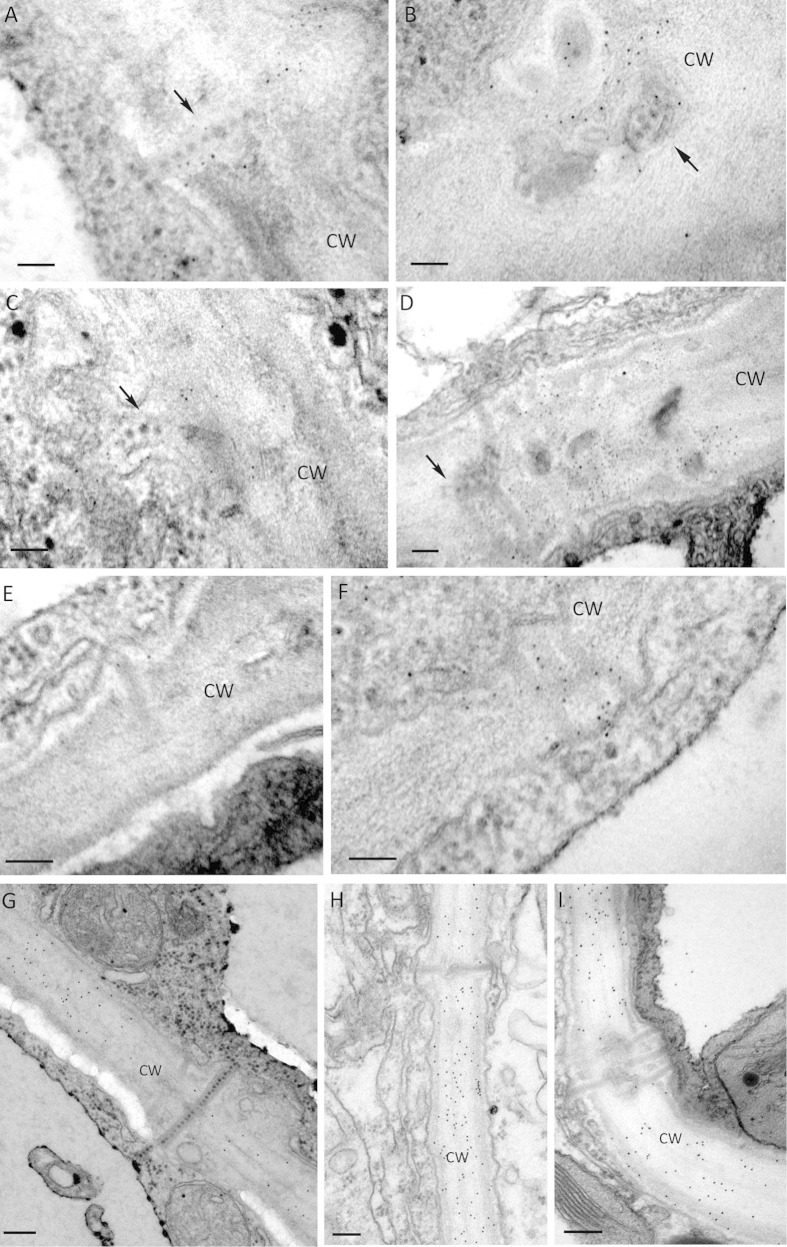
Immuno-gold labeling of the cell wall components callose and cellulose in a broad bean wilt virus 2 infected *Chenopodium quinoa* leaves. (**A–D**) Specific gold labeling of callose was observed on plasmodesmata (PD) associated with VP37-tubules. VLPs associated with the VP37-tubule are marked with an arrow. Bar = 100nm. (**E**) Negative labeling of callose on single-lined PD lacking VP37-tubules. Bar = 100nm. (**F**) Specific gold labeling of callose observed on branched PD lacking VP37-tubules. Bar = 100nm. (**G**) Positive labeling for cellulose location on normal cell wall (CW) and absence of cellulose on VP37-tubules. Bar = 200nm. (**H,I**) Labeling reveals the absence of cellulose on PD exhibiting a complicated and simplified structure. Bar = 200nm. The colloid gold particles represent callose localization were of 5 nm in size, while the gold particles represent cellulose localization were of 15 nm in size.

Callose (β-1,3-glucan) is a CW constituent important for the formation of PD, and plays an important role in alterations of PD structure, while cellulose is the major component of the CW not associated with PD^5^. Through analysis of the tomogram digital slices, modifications of the CW around PD that contain VP37-tubules could readily be detected by their smooth texture and transparent appearance (Fig. 3B–F, asterisk). By way of contrast, the normal CW was composed of short fibril-like structures with moderate electron density. Based on these observations, we investigated whether alteration of the PD induced by VP37-tubules was a result of modification of CW components. Using an antibody against callose or an enzyme-gold probe for detecting cellulose, immuno-gold labeling was performed on VP37-tubules that either contained or lacked PD. Due to the high amount of callose and cellulose in PD or in the CW, a preliminary experiment was performed for obtaining an appropriate antibody dilution. In order to observe a clear difference in the concentration of polysaccharide between PD and normal CW, a high dilution (200 fold) of both the antibody against callose and the cellulose probe was used in this study. Using this dilution of callose antibody we observed clusters of colloidal gold particles associated with the CW in the vicinity of PD that contained VP37-tubules (Fig. 4A–D). Specific labeling was detected on both single-lined (5 positive in 7 samples) (Fig. 4A,C) and branched PD (4 positive in 4 samples) (Fig. 4B,D) that contained VP37-tubules. As a control, immuno-gold labeling for callose deposition using the same antibody dilution, was also observed on PD lacking VP37-tubules. Of 66 single-lined PD, only 4 exhibited positive labeling for callose (6.1% of positive), indicating that the majority (93.9%) of single-lined PD lacked callose deposition (Fig. 4E). However, of the 92 branched PD, 72 exhibited positive labeling (78.3%) on the CW in the vicinity of PD (Fig. 4F). We interpret this to indicate that substantial deposition of callose at the CW occurs at branched PD, as opposed to single-lined PD. Furthermore, at a 200 fold dilution of the enzyme-gold cellulose probe, no gold labeling was detectable on PD that either contained or lacked VP37-tubules (Fig. 4G) and the non-VP37 tubule contained PD (Fig. 4H,I). Together, this indicates a reduction in cellulose at those locations. Thus, we can conclude that callosic deposition in PD containing VP37-tubules resembles that of branched PD rather than single-lined PD, and that callose deposition on the CW that has been structurally altered around PD or VP37-tubules is associated with cellulose reduction.

## Discussion

In this study we investigated the relationship between VP37-tubules and PD during BBWV 2 infection, using TEM methods, including conventional observation, 3D ET reconstruction and immuno-gold labeling. We observed that at the cytological level, association of VP37-tubules with PD brought about differential effects on each other. Different types of PD had different effects on the frequency of VP37-tubules on different cellular interfaces as well as between primary and secondary PD. In parallel, the association of VP37-tubules with PD correlated with structural alterations and CW modification of PD.

Our observation has provided some evidence for the existence of different types of tubules formed by the BBWV 2 MP. Elongation of the MP-derived tubule from the PD to the cytoplasm was not only found in BBWV 2 but also in CPMV^23^. Although the elongated part of tubule was free from the confines of the PD, the profile of the tubule in cytoplasm was the same as that in the PD. The observation that MP-derived tubules can extend from the PD into the cytoplasm is consistent with observations that MP-derived tubules can form on the surface of CPMV infected cowpea protoplasts, where the CW and PD do not exist^24^,^25^. The CPMV-MP tubule-like structure observed on the protoplast surface had a widened morphology and was packaged with multiple lines of virions^26^. Thus, in both BBWV 2 and CPMV MP-tubules containing virions maintain their intrinsic morphology with minimal alteration even in the absence of PD restriction. In BBWV 2, a tubule-like structure has also been observed on the surface of tobacco BY-2 protoplasts and insect *Trichoplusia ni* cells, in which only VP37 protein was expressed^19^. These reports imply that MP is the only viral component of the tubule, and that the PD is not required for MP-tubule assembly. Apart from tubule elongation, the other morphologies we observed have seldom been reported, in particular the widened tubules packaged with multiple lines of virions.

For ‘tubule-guided’ viral movement, there have been few reports related to the distribution and frequency of MP formed tubules. Results on the distribution and frequency of BBWV 2 MP-derived tubules may provide important information regarding the movement of viruses in different tissues. For example, the observation that MP-derived tubules are found in PD of phloem cells might imply that the long-distance movement of BBWV 2 occurs through the vascular bundle. In addition, observations that there were differences in the occurrence of MP-tubules in different tissues might imply that BBWV 2 moves with different efficiencies in those tissues. Moreover, our results show that there appears to be a preference for MP-tubule formation between single-lined and branched PD morphology, with a higher frequency in single-lined PD. For TMV, MP is located within secondary PD^14^ and functions to increase the SEL of PD only in epidermal cells at the leading edge of the lesions, but not in cells within center of the lesion^27^. From our observations of BBWV 2, it can be concluded that VP37-tubule generation can occur in both single-lined and branched PD, and possibly within primary and secondary PD. Unlike TMV, our results suggest that BBWV 2 MP can alter PD in systemically infected leaves, including epidermal, mesophyll and phloem tissue. From our results it appears as though expression of BBWV 2 MP results in a greater increase in the opening of PD than that observed for TMV MP. The selectivity of PD is continually changing with respect to the intercellular transport of different proteins^28^, but the role of the BBWV 2 MP-tubule in altering the SEL of PD for viral movement needs further study.

Our results revealed that the association of BBWV 2 MP-derived tubules with PD results in expansion of the PD. The DT-like structure apparent in PD that had no associated MP-derived tubules was lacking in PD that were associated with MP-derived tubules, as revealed by 3D ET. Cytoskeletal proteins such as actin and myosin, are components of the DT that are necessary for intercellular communication^2^,^29^. The disappearance DT in PD has also been reported during the formation of MP-tubules^15^, however without direct 3D structural evidence. Our results have confirmed the absence of DT in PD containing BBWV 2 MP-derived tubules. It is formally possible that components of the DT participate in the formation of VP37-tubules. However this still needs to be determined.

In response to viral infection, plant hosts use callose deposition at PD as a defense strategy to block the ability of a virus to move through the plant^5^,^9^. Intercellular movement of TMV and SMV, which do not induce tubule formation in PD, is however associated with host induced callose deposition^30^,^31^. In response to the deposition of callose, some viruses cause degradation of the callose to promote spreading of the virus through the plant. Transgenic tobacco engineered to silence the enzyme responsible for callose degradation (β-1,3-glucanase), accumulated callose and the SEL of PD was reduced, leading to a reduction in TMV infectivity^32^. Also, local expression of β-1,3-glucanase in tobacco enhanced symptoms typical of a TMV infection^33^. To date, little evidence has been provided for the deposition of callose during movement of a tubule-forming virus, such as BBWV 2. Our results provide evidence that plants, when infected with a tubule-forming virus, also induce the deposition of callose. With regard to callose deposition in PD associated with MP-derived tubules, we believe it has multiple functions. Callose plays an important role in the regulation of PD permeability as it is the material that can alter the CW as a result of reduced cellulose content^5^. During VP37-tubule formation in BBWV 2 infection, PD underwent obvious structural alterations, and callose deposition was observable. However, in virus-free controls we also observed callose deposition around secondary PD that did not contain tubules but exhibited obvious structural alterations. The pattern of callose deposition in these two cases looked very similar. It is therefore reasonable to suggest that callose deposition participates in the alteration of PD. To verify the exact function of callose deposition and reduced cellulose content in the movement of BBWV 2, we must examine whether callose degradation and cellulose reduction are associated with VP37-derived tubules. These studies are currently underway.

## Materials and Methods

### Virus and antibodies

The BBWV 2 virus (isolate PV131) used in this study was obtained from Prof. Vittoria Lisa (Istitio Di Fitovirologia Applicata, Italy). Natural host plant of *C. quinoa* was inoculated with BBWV 2 and maintained in at 26 °C in a greenhouse under natural light. The position of each leaf for sampling was coordinated from the source leaf, in which the secondary PD are distributed according to previous work on TMV MP^14^. *C. quinoa* mature leaves exhibiting symptoms typical of an infection, including crinkle, wilt and mosaic, were collected for investigation at 7 days post inoculation (dpi). Sampling for the TEM studies was performed three times.

For the immuno-gold labeling of cell wall material, a monoclonal antibody against callose (β-1,3-glucan) (Biosupplies, Australia) and a 5 nm IgG-gold conjugated secondary antibody (anti-mouse serum, Sigma, St. Louis, MO) were purchased. An enzyme-gold probe for detecting cellulose was also used in the labeling experiments, which was kindly provided by Prof. D.W. Hu^34^. Additionally, for immuno-gold labeling of virus particles, a polyclonal antibody against BBWV 2 virions and a 15 nm protein A-gold conjugated secondary antibody have been previously described^35^.

### TEM tissue preparation

Tissue preparation including fixing, dehydration and embedding were carried out following the described protocol^19^,^36^. Systemically infected leaves, and uninfected leaves from the same position, were harvested from three cultivar repeats. The symptomatic area of an infected leaf, and a similar area from an uninfected leaf were first cut into small pieces (1×1 mm) to facilitate permeation of the fixing agent. For ultra-structural observations, samples were first fixed using 2.5% glutaraldehyde for 4h and then 1% osmium tetroxide for 2h at room temperature. For immuno-gold labeling, samples were fixed using a mixture of 4% paraformaldehyde and 0.1% glutaraldehyde for 1h at 4 °C. Each fixation was followed by a 15 min rinse with 100 mM pH 7.0 sodium phosphate buffer (PBS) for three times to remove the fixative. Samples used for ultra-structural observations were dehydrated by a graded ethanol series (50%, 70%, 80%, 90%, 95% and 100%) followed by 100% acetone for 20 min each at room temperature. Samples were embedded into Epon 812 resin and polymerized by heating to obtain solid blocks. Samples used for immuno-gold labeling were dehydrated by graded ethanol series (30% and 50% at 4 °C for 0.5h each and 50%, 70%, 90% and 100% at −20 °C for 1h each). Embedding of these samples into K4M resin was achieved by ultra-violet light polymerization at −20 °C to preserve the immune activity. Ultra-thin sections (50–70 nm) of the sample blocks were cut by a Leica UC 6 microtome (Leica, Vienna, Austria) with diamond knife (Diatome, Switzerland). For cytopathology, sections were cut from the Epon resin blocks and placed onto 200 mesh copper grids. For gold labeling, sections were cut from the K4M and Epon 812 resin blocks and placed onto 150 mesh nickel grids.

### Immuno-gold labeling and TEM observation

Immuno-gold labeling of virus particles was performed using sections from K4M resin, which preserved antigenicity of the protein, but poorly preserved the fine structure of the tissue. Immuno-gold labeling of callose or cellulose was made using sections from Epon812 resin, which preserved both the polysaccharide antigenicity and fine structure. Sample grids were pre-treated with ddH_2_O for 5 min, followed by incubation in blocking buffer (EMS, Hatfield, PA) for 30 min at 37 °C. Sample grids were then incubated in primary antibody raised against either callose (200× dilution) or BBWV 2 virus particles (50× dilution) for 2h at 30 °C. Sample grids were washed three times with PBS and three times with ddH_2_O for 5 min each to remove excess antibody. Incubation of the sample grids with IgG-gold conjugated secondary antibody (100× dilution) was carried out for 2h at 37 °C. After washing the sample grids three times with PBS and three times with ddH_2_O for 5 min each, the antigen-antibody interaction was fixed in 4% paraformaldehyde for 5 min. Pre-immune serum was used as a negative control. For enzyme-gold labeling of cellulose sample grids were incubated with probe (200× dilution) for 30 min at 37 °C, followed by washing three times in ddH_2_O for 5 min each. Incubation of gold without probe was used as a negative control. Before labeling of polysaccharide, a preliminary experiment was carried out that demonstrated a 200 fold dilution of the antibody or probe was appropriate to distinguish differences in polysaccharide concentration on specific structures.

Before TEM observation, all sample grids were stained with uranyl acetate and lead citrate for 15 min each to increase image contrast. Sections for cytopathology and immuno-gold labeling were examined using an H-7650 TEM (Hitachi, Ibaraki, Japan) at 80kV of accelerating voltage. Regions of the VP37-tubule were photographed using a Gatan 830 CCD camera (Gatan, CA, USA).

### Frequency study of VP37-tubule occurrence and distribution

No existing method was available for the study of VP37-tubule frequency. A PD frequency (F_PD_) study had been reported^37^, but F_PD_ could not be used to represent tubule frequency because only a portion of the PD contained VP37-tubules. Also, a parameter consisting of tubule number per CW length was not comparable because F_PD_ varied between different tissues. Therefore, to exclude the variation in PD occurrence we developed the occurrence frequency (Occu-F_tubule_) to describe the occurrence of VP37-tubules in PD. This is calculated from the tubule number divided by the PD number (N_tubule_/N_PD_) and describes the occurrence of VP37-tubules in PD. In addition, we developed the distribution frequency (Dist-F_tubule_), which is calculated by Occu-F_tubule_/F_PD_ to describe the distribution of VP37-tubule on the CW. The two parameters, Occu-F_tubule_ and Dist-F_tubule_, were used to indicate VP37-tubule frequency. All fundamental parameters including CW length, PD number and VP37-tubule number in this study were measured under TEM. Two kinds of image were photographed. Large field (under 0.7k magnification) images were used for measurements of CW length (L_CW_) and number counting, while high-resolution images (under 50 k magnification) were used for ultra-structural details. L_CW_ measuring was performed using the software of pathology image digital processing system ‘JD801’ (JEDA company, Nanjing, China). For a VP37-tubule associated with PD to be scored as positive, we set the parameter that the tubular structure had to package a string of at least two virus particles. A frequency comparison was made between different cellular interfaces as well as between single-lined and branched PD. A total of 40 sections from five blocks (eight sections in each block) were made. However, the small number of VP37-tubules in one TEM field sometimes led to a zero value for N_tubule,_ which prejudiced the average statistic. Therefore, a larger sample capacity was needed. For the sampling of one section, we summed the values from 20 randomly selected TEM fields, which guaranteed that a relatively large number of VP37-tubules were counted for statistical analysis. Each sample field consisted on average, of 10–15 mesophyll cells (MC) or three to four epidermal cells (accompanied with some MCs) or 30–40 phloem cells (accompanied with some MCs). Thus, in total each sampling (20 fields) consisted of approximately 120–140 cells. This was repeated three times using different cultivars embedded in different resin blocks, and the frequency values calculated as an average of these replicates.

### Electron tomography (ET)

For ET reconstruction, sections (100–120 nm) were cut from the Epon resin blocks using a UC6 tomogram and placed onto 100 mesh carbon covered copper grids. 15 nm colloidal gold particles (Sigma, St. Louis, MO) were applied to the sample grids for 5 min, and then stained as described above. This non-covalent binding of gold particles to sections was used as a fiducial marker for facilitating tomography alignments. A four series image of the VP37-tubule and a one series image of normal PD were collected by a JEM-2010 TEM (JEOL, Tokyo, Japan) at 200kV of accelerating voltage and recorded by a Gatan 832 CCD (size 2.7 k × 4 k) camera (Gatan, CA, USA). For each series, the TEM specimen stage was moved over a ±60° range of tilt angles with 2° intervals^38^. On tilting, images were consistently photographed with the defocus value of 0.2 μm at 15k magnification under the beam density of 26 e/sÅ^2^.

Image processing, alignment and reconstruction were operated in the IMOD ‘etomo’ program^38^ following the procedures outlined in the software manual. For fine alignment, twenty gold particles were used as the fiducial markers for each tilt series to obtain a ‘residual error’ value of less than 0.3. The final three-dimensional (3D) tomograms for each tilt series were generated by combining images using an Å/pix size of 8.74 (compression parameter of ‘binning = 2’) in the ‘etomo’ program. Dimension measurements, model segmentation and surface rendering were carried out by the command ‘3dmod’ of the IMOD program^39^. Visualization of the model was performed using the ‘Chimera’ program^40^.

## Additional Information

**How to cite this article**: Xie, L. *et al.* Mutual association of *Broad bean wilt virus* 2 VP37-derived tubules and plasmodesmata obtained from cytological observation. *Sci. Rep.* **6**, 21552; doi: 10.1038/srep21552 (2016).

## Figures and Tables

**Table 1:** Numbers of VP37-tubules (sum total of 155) observed on different cellular interfaces and in different tissues.

	MC-MC*	EC-EC	EC-SGC	PPC-PPC	BSC-BSC
Cellular interface	124/155, 80%	19/155, 12.3%	2/155, 1.3%	7/155, 4.5%	3/155, 1.9%
Tissue	Mesophyll 80%	Epidermis 14.6%		Phloem 6.4%	

^*^MC: mesophyll cell, EC: epidermal cell, SGC: safe guard cell, PPC: phloem parenchyma cell, BSC: bundle sheath cell.

**Table 2:** Frequency of VP37-tubules associated with Plasmodesmata (PD) in different tissues.

	L_CW_(μm)*	N_PD_	N_tubule_	F_PD_	Occu-F_tubule_	Dist-F_tubule_
Epidermis	563 ± 13.748	59.667 ± 5.033	6 ± 1	0.106 ± 0.007	0.100 ± 0.008	0.945 ± 0.017
Mesophyll	2944 ± 30.806	368.333 ± 12.220	15.667 ± 1.155	0.125 ± 0.003	0.042 ± 0.003	0.340 ± 0.016
Phloem	2474.667 ± 41.356	160.667 ± 3.786	1.333 ± 0.577	0.065 ± 0.002	0.008 ± 0.004	0.128 ± 0.056

^*^The parameters of cell wall (CW) length (L_CW_), PD number (N_PD_) and VP37-tubule number (N_tubule_) were measured using images obtained by transmission electron microscopy (TEM). PD frequency (F_PD_) was then calculated from N_PD_/L_CW_, VP37-tubule occurrence frequency (Occu-F_tubule_) from N_tubule_/N_PD_ and VP37-tubule distribution frequency (Dist-F_tubule_) from Occu-F_tubule_/F_PD_. For all tissue types, the average frequency value (F) was calculated from three independent experiments.

**Table 3:** Frequency of VP37-tubules associated with single-lined and branched Plasmodesmata (PD).

LCW(μm)*	morphology	N_PD_	N_tubule_	F_PD_	F_PD_ ratio	Occu-F_tubule_	Occu-F_tubule_ ratio
5982 ± 82.245	single-lined	191 ± 16.523	11 ± 1.155	0.032 ± 0.002	1 : 2.062	0.056 ± 0.001	1.806 : 1
	Branched	398 ± 8.963	12 ± 1.155	0.066 ± 0.002		0.031 ± 0.004	

^*^The parameters of CW length (L_CW_), PD number (N_PD_) and VP37-tubule number (N_tubule_) were measured using images obtained by transmission electron microscopy (TEM). Frequencies were calculated by using PD frequency (F_PD_) derived from N_PD_/L_CW_, and VP37-tubule occurrence frequency (Occu-F_tubule_) derived from N_tubule_/N_PD_. A comparison between single-lined and branched PD is indicated as the F_PD_ ratio (F_PD_ single-lined)/(F_PD_ branched), or Occu-F_tubule_ ratio (Occu-F_tubule_ single-lined)/(Occu-F_tubule_ branched).
